# Characterization of Partially Covered Self-Expandable Metallic Stents for Esophageal Cancer Treatment: *In Vivo* Degradation

**DOI:** 10.1021/acsbiomaterials.0c01773

**Published:** 2021-03-12

**Authors:** Paulina Chytrosz, Monika Golda-Cepa, Janusz Wlodarczyk, Jarosław Kuzdzal, Miroslawa El Fray, Andrzej Kotarba

**Affiliations:** † Faculty of Chemistry, Jagiellonian University, 31-007 Kraków, Poland; ‡ Department of Thoracic and Surgical Oncology, Jagiellonian University Medical College, John Paul II Hospital, 30-387 Kraków, Poland; § Department of Polymer and Biomaterials Science, West Pomeranian University of Technology, 70-310 Szczecin, Poland

**Keywords:** esophageal stent, biomaterial, polyurethane, *in vivo* degradation, esophagus, esophageal cancer

## Abstract

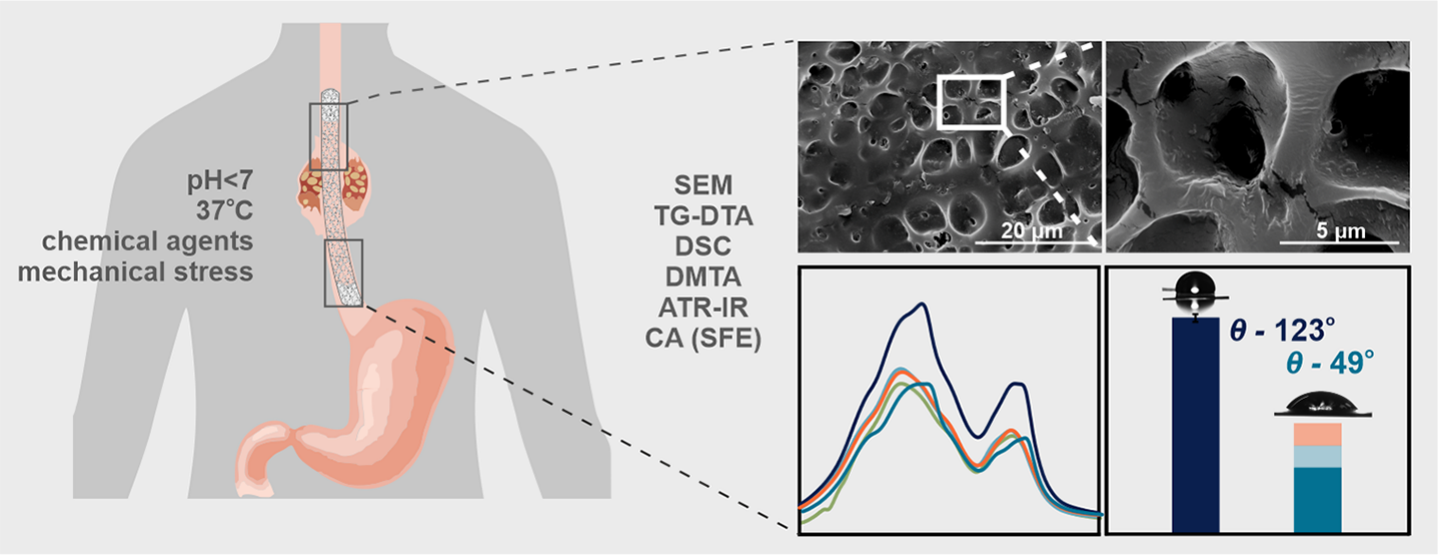

Partially covered self-expandable metallic esophageal stent (SEMS) placement is the most frequently applied palliative treatment in esophageal cancer. Structural characterization of explanted 16 nitinol-polyurethane SEMS (the group of 6 females, 10 males, age 40–80) was performed after their removal due to dysfunction. The adverse bulk changes in the polymer structure were identified using differential scanning calorimetry (DSC), differential mechanical thermal analysis (DMTA), and attenuated total reflectance infrared spectroscopy (ATR-IR) and discussed in terms of melting point shift (9 °C), glass-transition shift (4 °C), differences in viscoelastic behavior, and systematic decrease of peaks intensities corresponding to C–H, C=O, and C–N polyurethane structural bonds. The scanning electron and confocal microscopic observations revealed all major types of surface degradation, i.e., surface cracks, peeling off of the polymer material, and surface etching. The changes in the hydrophobic polyurethane surfaces were also revealed by a significant decrease in wettability (74°) and the corresponding increase of the surface free energy (31 mJ/m^2^). To understand the *in vivo* degradation, the *in vitro* tests in simulated salivary and gastric fluids were performed, which mimic the environments of proximal and distal ends, respectively. It was concluded that the differences in the degradation of the proximal and distal ends of prostheses strongly depend on the physiological environment, in particular stomach content. Finally, the necessity of the *in vivo* tests for SEMS degradation is pointed out.

## Introduction

Every sixth death in the world is due to cancer, the second leading cause of death.^1^ Esophageal cancer is one of the most commonly diagnosed cancers and the sixth leading cause of cancer-related mortality.^2^ Due to either a progressive tumor stage or weak physical conditions, the majority of patients are not eligible for curative treatment.^3^ The most important aspect of esophageal cancer is dysphagia, which is a late symptom of esophageal cancer, leading to weight loss, eating disorders, and related metabolic deficiencies; thus, to improve the quality of patients’ life, a palliative treatment must be applied.^4^

Stent treatment is well documented and commonly applied on a large scale for palliative treatment of dysphagia and fistula caused by advanced esophageal cancer. Clinical research confirmed that esophageal stent implantation has satisfying results, leading effectively to patients’ relief.^5^ The esophageal stenting is a relatively easy method, used for the first time in 1885. Since then, rapid development due to the advances in endoscopy has been observed.^6^

Nowadays, esophageal stents consist of two main components of different functions: nitinol mesh (superelastic shape memory alloy for expansion) and polymeric membrane (biocompatible cover), schematically presented in Figure SI.1. Such composite construction combines the advantages of the mechanical performance of metal alloy and elastic polymeric barrier, preventing tumor overgrowth.^7^ Self-expandable metal stents (SEMSs) are available in three variants, uncovered, partially covered, and fully covered with flanges at both ends, resulting in a “dog bone” shape.^8^ The greatest benefit of using SEMSs is their sufficient elasticity to be compressed for delivery and expanded after implantation in the target area within 24 h at the patient’s body temperature.^9^

Although the stent implantation procedure is rather simple and limits surgical invasion during stenting, there is still a risk of life-threatening complications. The most frequent postoperation complications are prosthesis migration, esophageal perforation, and overgrowth of the stent by the granulation tissue (hyperplastic tissue reaction). Moreover, there is a possibility of more serious consequences such as hemorrhage and fistulas between the esophagus and the bronchial tree or mediastinum.^4,10−12^ The factors causing the complications are poorly understood; however, despite the side effects, stenting still ensures improvement in the quality of life of palliative patients.

As suggested by producers, the maximum residence time of the prosthesis is 3 months. However, due to the above-mentioned complications, it is unachievable in many cases. A new approach in improving the quality of palliative treatment can be provided by local heat treatment using a nanofunctionalized SEMS^13^ or functionalization of the SEMS polymeric barrier with chemotherapeutic agents (docetaxel, paclitaxel, fluorouracil), antibiotics (doxorubicin), or radiotherapeutic agents (iodine-125, holmium-166).^14−18^ It is also worth mentioning the new generation of stents covered with a biodegradable polymer, which can be additionally equipped with a drug-eluting function. Some of their successful applications include covering a metal stent with a polycaprolactone (PCL)-based fibrous membrane containing paclitaxel.^19^ Such an approach allows in-site chemotherapy and effectively reduces the risk of restenosis.^20^

Polyurethanes (PUs), due to their specific physicochemical properties,^21−23^ have a broad gamut of medical applications in various fields including coating technology (coatings of orthopedic metal implants, wound dressings^24^), bulk biomaterials (middle ear implants),^25^ and scaffolds (orthopedic and cardiovascular).^26,27^ Polyurethanes are also suitable for applications as cardiovascular,^28^ neurological,^29^ and esophageal stents.^30^ The polymeric membranes made of polyurethanes are used for over 25 years as short-term biomedical devices.^31,32^ The three principal components of PU include polyol, diisocyanate, and cross-linker between which a urethane linkage (−NHCOO−) is formed. There is a variety of possible polyols (over 500 commercially used) that play an important role in controlling the characteristics of the PUs, such as physicochemical and mechanical properties.^33^ The PUs used as biomaterials are polyether- and polyester-based, and their choice depends on the application site. Polyether-based PUs are flexible and resistant to hydrolysis but have low oxidative and thermal stabilities, while polyester-based PUs have greater mechanical strength and heat resistance, though susceptible to hydrolytic degradation.^34^

To improve the polyurethane performance, intense studies are carried out, e.g., to enhance hemocompatibility and antibacterial activity. Two main paths of the PU surface modification are focused on the implementation of substances with a specific function (e.g., antibiotics, metallic nanoparticles, growth factors, natural polymers), or generation of active groups on polymeric surfaces by plasma treatments, flame treatment, UV irradiation, and chemical grafting.^35,36^

The polyurethane membranes of esophageal stents that prevent tumor overgrowth should be stable and ensure reliable support against dysphagia, and in long-term use, a significant loss of biostability is observed.^37,38^ The degradation is caused by the very aggressive environment of the body fluids, living tissues, and a combination of physical and chemical processes at the implant–body interface such as hydrolysis, oxidation stress, environmental stress cracking, and calcification.^39−41^ Crack formation and propagation on the surface of the polyurethane is mainly caused by environmental stress. In general, the breaks in the polymer chain caused by the synergistic effects of chemical degradation and mechanical stress generate microscopic defects, leading to the formation of cracks and their propagation on the surface.^37^ Such degradation of PU-based implants results in a decrease in mechanical strength. The process of *in vivo* degradation depends on many factors, i.e., it is assumed that both the environmental factors of patients (cancer stage and aggressiveness, health/condition) and the treatment (chemotherapy, radiotherapy) play an important role here. Unfortunately, the exact causes are unknown, because no scientific attempts have been made to assess them so far, and there are no systematic scientific reports, especially for *in vivo* conditions.

The aim of this study was to compare the reference (as received from the producer) and implanted stents (removed from patients after 1–6 months) in terms of surface and bulk changes in the polyester-based polyurethane membranes. Such an approach requires application of a broad range of physicochemical methods dedicated to the thorough characterization of polymeric materials such as differential scanning calorimetry (DSC), differential mechanical thermal analysis (DMTA), attenuated total reflectance infrared spectroscopy (ATR-IR), scanning electron microscopy (SEM), confocal microscopy (CM), and contact angle (CA) measurements.

## Materials and Methods

### Patients

Sixteen patients (10 men, 6 women in the age group of 42–82, average age 59) were palliatively treated for esophageal tumor by the placement of self-expandable polyurethane stents (SEMS, made of a nitinol mesh and covered with a polymeric membrane with flanges at the proximal and distal ends.) Two main treatments were applied, chemotherapy and radiotherapy. SEMSs were removed from living patients by endoscopy due to total dysphagia caused by an overgrowth of noncancerous granulation above or below the implanted prosthesis or during esophageal resection. Esophagus resection was not a routine surgical procedure for patients qualified to palliative treatment. A detailed summary of patients’ information (treatments, re-treatments, and complications) is listed in Table 1.

**Table 1:** Basic Information on the Investigated SEMS

		treatment				
patient	gender	stenting	chemotherapy	radiotherapy	complication	residence time (days)
1	M	+	+	+	bronchus fistula	35
2	F	+	+	+	granulation	66
3	F	+			granulation	153
4	M	+	+	+	migration	68
5	M	+	+	+	granul. empyema	91
6	M	+	+	+	none	93
7	F	+	+	+	none	98
8	M	+	+	+	granulation	82
9	M	+	+	+	granulation	45
10	M	+	+	+	bronchus fistula	38
11	M	+	+	+	none	101
12	F	+	+	+	granulation	31
13	M	+	+	+	bronchus fistula	81
14	F	+	+	+	granulation, dysphagia	65
15	M	+	+	+	granulation, dysphagia	48
16	F	+	+		granulation	62

### Samples

The removed esophageal stents were cut into three parts (proximal, distal, and middle). Each one was stored separately. Prior to the investigations, for thorough cleaning from adsorbed organic residues (mainly proteins and bacterial biofilm), the samples were placed in a beaker with distilled water and kept in an ultrasonic cleaner for 10 min. After the cleaning, the samples were air-dried.

### Material Characterization

#### Differential Scanning Calorimetry (DSC)

The changes in bulk properties of polyurethane were analyzed using a DSC 821e Mettler Toledo apparatus. The experiments were performed in the temperature range of 25–600 °C with a heating rate of 10 °C/min at an Ar flow.

#### Dynamic Mechanical Thermal Analysis (DMTA)

A Q800 DMA (TA Instruments, New Castle, DE) apparatus operating in a tensile mode was used to determine the storage modulus (*E*′), loss modulus (*E*″), and the tangent of the phase angle (tan δ). The glass-transition temperature (*T*_g_) was taken as the maximum of tan δ and the maximum of loss modulus, *E*″. The relaxation spectrum was scanned from −70 to 150 °C, at a frequency of 1 Hz and a heating rate of 3 °C/min.

#### Attenuated Total Reflectance Infrared (ATR-IR) Spectroscopy

ATR-IR spectroscopy was performed to study the bulk changes in the stents’ polymeric component. The measurements were carried out on a Nicolet 6700 Thermo Scientific with a diamond crystal. The spectra were acquired in the range of 4000–600 cm^–1^, and for each sample, the result is an average of 64 scans.

#### Scanning Electron Microscopy (SEM)

The images of eroded samples’ surfaces for the polymer and nitinol were taken using a Hitachi S-4700 scanning electron microscope. The polyurethane samples were coated with Au prior to the observations due to their nonconductive nature.

#### Confocal Microscopy

The images of polymeric samples to measure their roughness were taken using an Olympus Lext OLS4000 confocal microscope (magnification, 10×; image size, 1280 × 1281 μm^2^). Surface roughness was calculated based on the formula for *R*_a_, which is the arithmetic average of the absolute values of the profile height deviations from the mean line, recorded within the evaluation length. The typical image used for evaluation of surface roughness is presented in Figure SI.2.

#### Contact Angle (CA) Measurements and Surface Free Energy (SFE) Calculations

The changes in the hydrophobicity caused by human body fluids were followed by contact angle measurements, using a Surftens universal instrument (OEG GmbH). Static contact angles of water and diiodomethane were calculated using software Surftens 4.3. For each sample, at least three 1 μL droplets of water and diiodomethane were applied, respectively. The mean value was the average over 10 independent measurements. SFE calculations, based on contact angle values of water and diiodomethane, were performed using the standard Owens–Wendt method dedicated to polymeric surfaces. The method of calculation and equations are described in detail in our previous work.^42^

#### *In Vitro* Aging Test of Polyurethane Membrane

To get more in-depth insight into polyurethane membrane degradation, *in vitro* tests were performed. For the best imitation of the stent environment in the human body (for the proximal and distal ends), two types of body fluids were used: artificial saliva and simulated gastric fluid. A modified Fusayama’s artificial saliva (AS) was used at 37 °C (pH 6), with the following chemical composition: NaCl (0.4 g/L), KCl (0.4 g/L), CaCl_2_·2H_2_O (0.795 g/L), Na_2_S·9H_2_O (0.005 g/L), NaH_2_PO_4_·2H_2_O (0.69 g/L), and urea (1 g/L).^43^ Simulated gastric fluid without pepsin, 0.2% (w/v) NaCl, in 0.7% (v/v) HCl, pH 1.5, was prepared.^44^*In vitro* experiments were performed for 62 days at 37 °C in an incubator with gentle shaking (60 rpm). The simulated body fluids were replaced every 7 days. The samples were collected for 7, 14, 21, and 62 days.

## Results and Discussion

**Figure 1:**
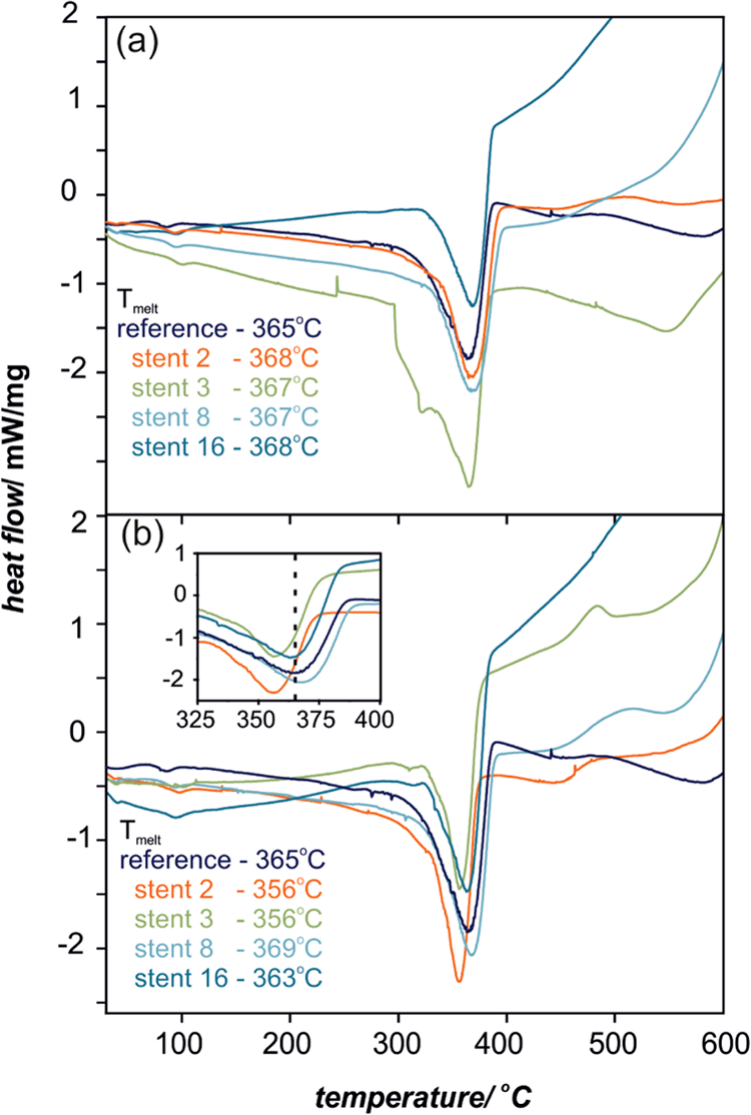
DSC profiles and the characteristic *T*_melt_ values for proximal (a) and distal (b) ends of SEMS polyurethane cover. The inset in (b) shows a narrow range of *T*_melt_ for the distal end of the prostheses.

**Figure 2:**
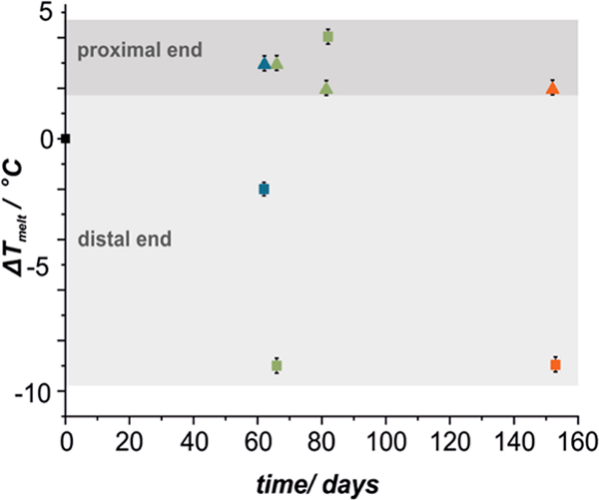
Changes of *T*_melt_ with implantation time for proximal (▲) and distal (■) ends of SEMS polyurethane (colors represent patients’ treatment: blue, chemotherapy; green, chemo- and radiotherapy; orange, palliative treatment only, i.e., stenting).

The polyurethane prosthesis membranes were thoroughly characterized in terms of bulk and surface properties to determine the effect of cancer therapy on their structure. Bulk changes in the polyurethane structure were investigated using DSC. Measurements were performed for prostheses exposed to various patient treatments: stenting, chemotherapy, chemotherapy, and radiotherapy. The DSC curves for representative samples are shown in Figure 1a for the prosthesis proximal end and in Figure 1b for the distal end. The characteristic parameter that indicates the changes in the polymer bulk properties is melting temperature (*T*_melt_); in the case of the reference sample, *T*_melt_ = 365 °C. For the implanted samples, significant differences in *T*_melt_ for proximal and distal ends were observed (for details, see the inset in Figure 1). In general, the observed trend for the proximal end is increase in *T*_melt_ in the narrow range of 367–368 °C, while for the distal end, the values are spread in the larger range of 356–369 °C, as shown in Figure 2 (the error bars correspond to 0.2 °C). The temperature shift (∼9 °C) for stent 2 and stent 3 points out significant bulk changes in the polymeric structure. It is worth mentioning that these samples are representatives of the material, which underwent combined chemo- and radiotherapy (stent 2) and the longest implantation time of 153 days (stent 3). The results also indicate that the distal end of the prosthesis, exposed to harsher environment conditions (i.e., stomach content), is more damaged in contrast to the proximal end, where the changes in the polymeric structure are minor. This also clearly illustrates that the chemistry of degradation processes is different on both prosthesis ends, i.e., the increase and decrease in *T*_melt_ of proximal and distal ends, respectively. Since melting temperature is characteristic of the structure of polymeric material, the degradation processes of the examined materials are not confined to the surface but advanced into the bulk. For detailed in-depth characterization of the polymer *in vivo* degradation and difference between distal and proximal sides, dynamic mechanical thermal measurements were performed. DMTA allowed for a detailed analysis of the temperature-dependent viscoelastic properties and gave insights into the mechanical behavior of the investigated polymeric membranes.

**Figure 3:**
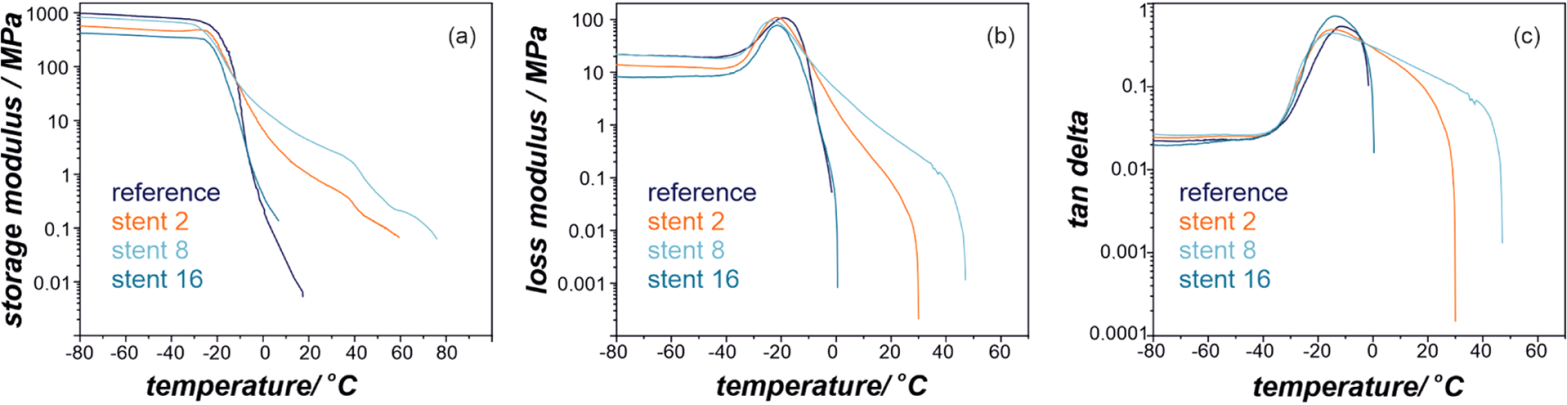
Storage modulus, *E*′ (a), loss modulus, *E*″ (b), and tan δ (c) profiles for the proximal end of SEMS polyurethane covers.

**Figure 4:**
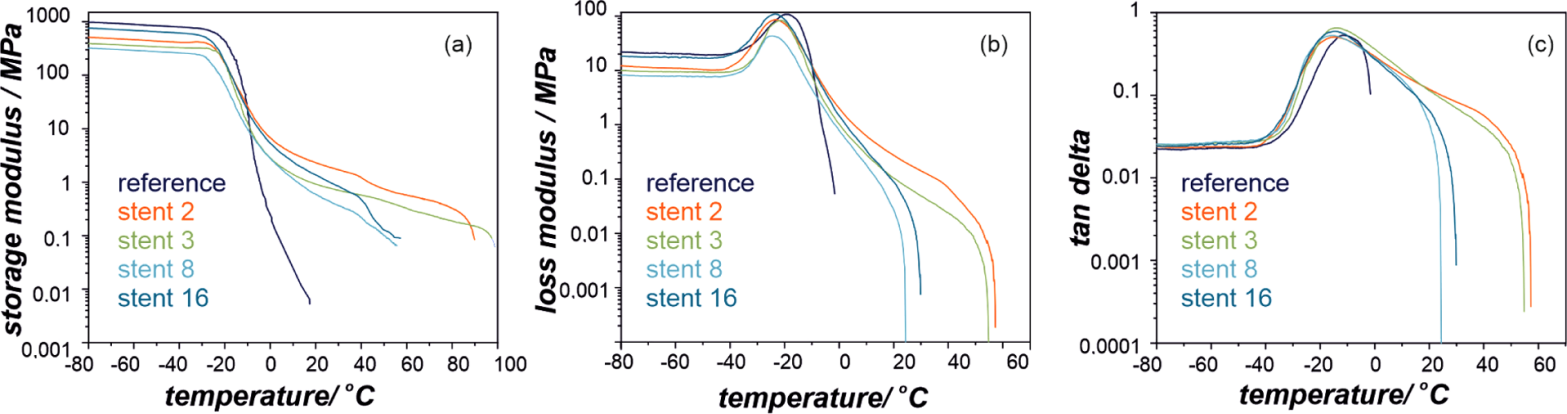
Storage modulus, *E*′ (a), loss modulus, *E*″ (b), and tan δ (c) profiles for the distal end of SEMS polyurethane covers.

The DMTA results are summarized in Figures 3 and 4, where values of the storage modulus, *E*′, are presented. The *E*′ values (Figures 3a and 4a) represent elastic behavior in viscoelastic polymers and indicate a clear difference in the elasticity with the change of various patients’ treatment: stenting, chemotherapy, and combined chemo- and radiotherapy. These changes can also be seen in *E*′ for proximal (Figure 3a) and distal (Figure 4a) ends of the polymeric stents cover. The highest difference in *E*′ between reference and implanted materials in the low-temperature region (before the sharp decrease around the glass-transition temperature, *T*_g_) has been noted for explants after stenting and chemotherapy for the proximal end, while for the distal end, the combination of stenting, chemotherapy, and radiotherapy had the most pronounced effect. Interestingly, all explanted stents from the distal region revealed typical for thermoplastic polyurethanes rubbery plateau, not observed for the reference material. This can indicate significant changes in the microphase separation and crystalline structure changes as these materials (stents 2 and 3, Figure 3a) show very distinct flow properties (melting transition). Figures 3b,c and 4b,c show storage modulus, *E*″, and tan δ curves, respectively. The maximum of *E*′ and tan δ value is often referred to *T*_g_ in thermoplastic polyurethane elastomers, and the values of the maxima of these transitions are summarized in Table 2. Again, the largest differences in *T*_g_ (taken as the maximum of *E*″) between the reference material and explanted PU has been found for the distal end after combined stenting, chemotherapy, and radiotherapy (Figure 4b). A significant broadening of the tan δ curves for explants after such treatment clearly indicates that changes in materials’ microstructure have occurred, which is in line with the DSC results.

**Table 2:** Glass-Transition Temperature (*T*_g_) of Polymeric Materials Covering Stents as Determined from DMTA as the Maximum of *E*″ or the Maximum of tan δ^a^^a^

sample code	*T*_g_ (max *E*″) (°C)	*T*_g_ (max tan δ) (°C)
stent 2 (66 days; S, R, C)	–21.62 (−23.02)	–14.81 (−14.85)
stent 3 (153 days; S)	– (−21.51)	– (−14.25)
stent 8 (82 days; S, R, C)	–23.21 (−24.45)	–15.23 (15.18)
stent 16 (62 days; S, C)	–21.31 (−23.44)	–13.90 (−14.40)
reference	–19.03 (−19.03)	–11.45 (−11.45)

a *T*_g_ (max *E*″), glass-transition temperature taken from the maximum of storage modulus, *E*″; *T*_g_ (max tan δ), glass-transition temperature taken from the maximum of tan δ. The numbers outside parentheses refer to the proximal end of the stent; the numbers within parentheses refer to the distal end of the stent. S, stenting; C, chemotherapy; R, radiotherapy.

**Figure 5:**
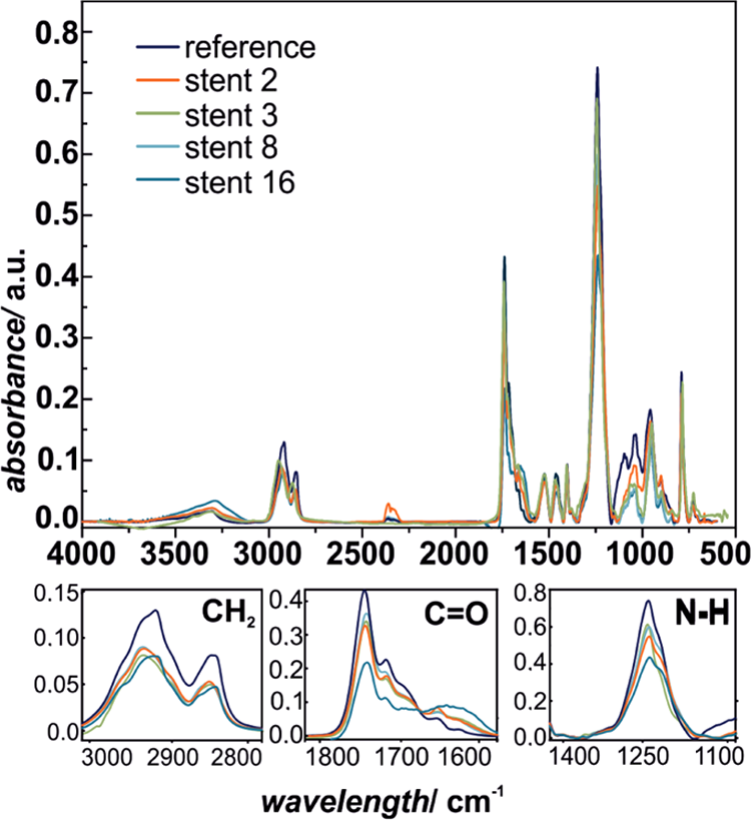
ATR-IR spectra of SEMS polyurethane covers: reference sample and selected stents (proximal end).

**Figure 6:**
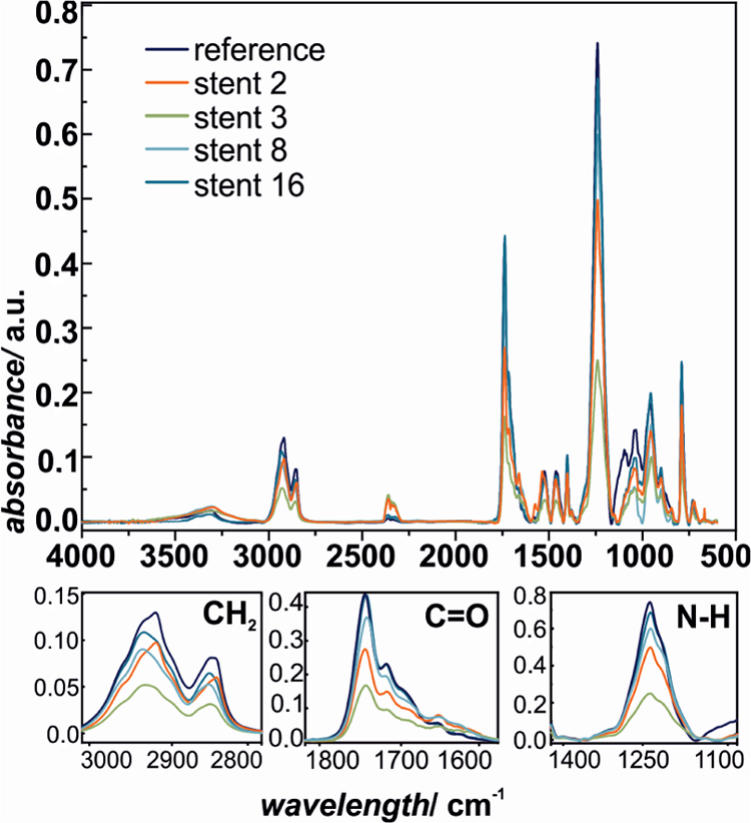
ATR-IR spectra of SEMS polyurethane covers: reference sample and selected stents (distal end).

The more in-depth insight into the chemical changes in the polyester polyurethane molecular structure can be obtained by infrared spectroscopy; therefore, the examined prostheses were investigated using ATR-IR, and the representative spectra are presented in Figures 5 and 6. The characteristic maxima for polyurethane are observed at 2930 and 2858 cm^–1^ (asymmetric CH_2_ stretch vibration),^45^ 1738 and 1716 cm^–1^ (C=O stretch vibration),^45,46^ 1524 cm^–1^ (coupling N–H blending vibration with C–N stretching vibration),^46^ 1463 and 1403 cm^–1^ (CH_2_ bending vibration),^47^ 1244 cm^–1^ (amine III vibration, C–N), 1044 cm^–1^ (antisymmetric C–O–C),^47^ 955 cm^–1^ (C–H benzene ring out-of-plane bending),^48^ and 790 cm^–1^ (amine IV vibration).^49^ Although the overall IR spectra are essentially similar, the detailed analysis of the three regions assigned to −CH_2_ (3000–2800 cm^–1^), C=O (1800–1600 cm^–1^), and C–N (1300–1150 cm^–1^) shows systematic changes in the characteristic bond absorption intensities, as presented in the bottom panels of Figures 3 and 4, respectively. This indicates that the extensive degradation process of the polymer took place upon implantation in the case of all of the investigated samples and the damage of distal ends is much greater compared to the proximal end. The ATR-IR results are in line with the *T*_melt_ values obtained using DSC, since again for the prosthesis numbers 2 and 3, the changes were the most pronounced in terms of all of the observed chemical groups, mainly CH_2_, C=O, and C–N.

**Figure 7:**
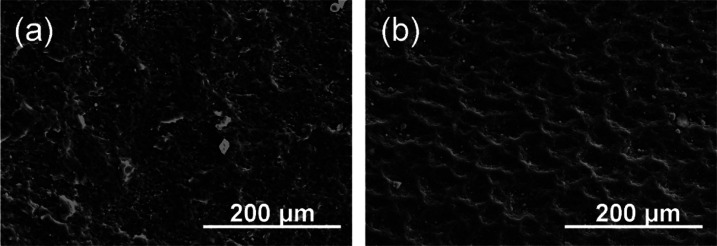
Representative images of reference sample morphology of SEMS polyurethane cover: outer side (a) and inner side (b) of the stent.

**Figure 8:**
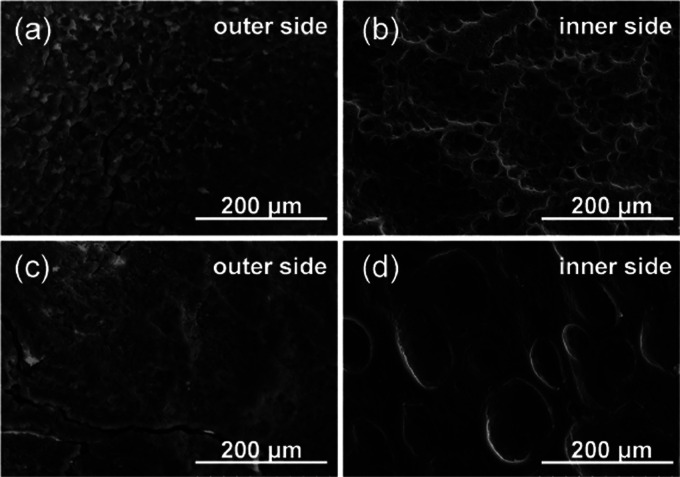
Representative SEM images of SEMS polyurethane covers with visible surface damages appearing after 9–12 weeks implantation time.

**Figure 9:**
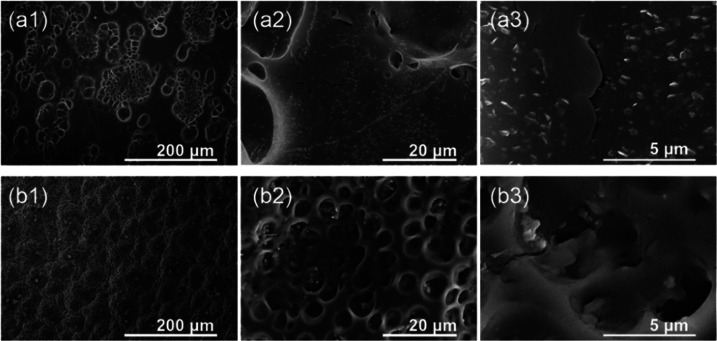
Representative SEM images illustrating the surface degradation (prosthesis 16) for the proximal end of the esophageal implant (a) and for parallel *in vitro* experiment in artificial saliva for 2 months (b).

**Figure 10:**
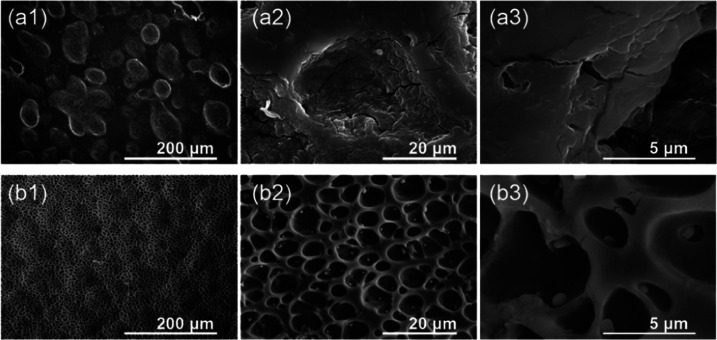
Representative SEM images illustrating the surface degradation (prosthesis 8) for the distal end of the esophageal implant (a) and for *in vitro* experiment in simulated gastric fluid for 2 months (b).

All of the methods (DSC, DMTA, and ATR-IR) complementarily revealed advanced changes in material properties. These adverse bulk changes are the effect of the surface damages caused by the aggressive environment of the body fluids and living tissues, which penetrate the material; therefore, scanning electron and confocal microscopic observations were performed to illustrate the changes in surface morphology. The representative images of the investigated samples are presented in Figures 7–10.

The SEM images illustrate the morphological differences in polymeric coating between the outer (dedicated to contact the tissue) and inner sides, as presented in Figure 7a,b. The outer surface of the polymer is smoother than the inner side, which has micro- (30–50 μm) and submicropores (>10 μm). On the top of each side, granulated particles (white spots) are observed, which were identified by independent X-ray fluorescence (XRF) measurements as magnesium oxide particles.

After implantation, the topography of the surface dramatically changed. The typical examples of prostheses morphologies are shown in Figure 8, where different types of surface damages can be observed. Placing the implants in the human body led to several processes resulting in severe changes in the surfaces of the implant. Indeed, in Figure 8, cracks (Figure 8a,c), peeling (Figure 8a), and material surface transformation from micron and submicron pores (Figure 8b) to ∼100 μm craters (Figure 8d) in the polymeric surface can be observed, indicating that the destruction processes are quite extensive. This is particularly visible for the inner side of the stents.

During the implantation, the surface of the polymeric component was coated with various human proteins and macrophages, which can strongly enhance the surface degradation process of biomaterials by the extensive release of oxygen free radicals.^50−52^ In Figures 9 and 10, more in-depth morphology analyses for prostheses 16 and 8 are presented, respectively. For combined chemo- and radiotherapy (stenting for 62 days), as well as prolonged residence in the body (82 days), all major types of surface degradation can be distinguished, i.e., polyurethane surface cracks, peeling off of the material, and surface etching. Thus, it can be suggested that the degradation processes taking part at the polymeric surface are interlaced.

To eliminate the effect of biological moieties we performed *in vitro* degradation tests of polyurethane membrane in simulated salivary and gastric fluids, representing the environment where the proximal and distal ends are placed. The corresponding images are presented together with *in vivo* results (Figures 9 and 10; see Figures SI.3–SI.6 for images from more systematic studies). The images clearly illustrate that the simulated body fluids alone already cause the degradation of the material. The walls of the pores become thinner (Figures 9b and 10b), whereas the longer incubation time results in characteristic cracks. It can also be noted that on the polymeric surface, the crystalline precipitates are formed, which contain the typical ions presented in the simulated saliva (Na^+^, K^+^, Ca^2+^, PO_4_^3–^, Cl^–^). It is mostly visible in the case of artificial saliva (pH 6) since the high ionic strength of the gastric solution (pH 1.5) hinders the formation of the precipitated salts.

**Figure 11:**
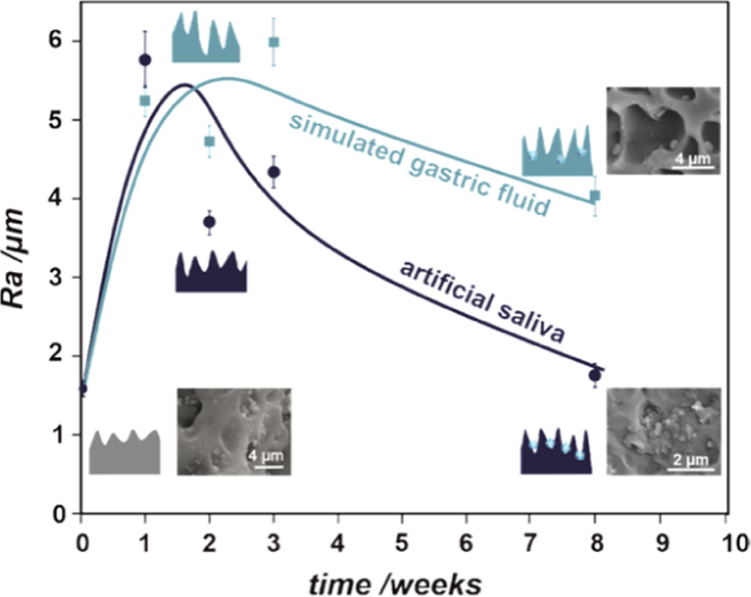
Surface roughness (parameterized by *R*_a_) as a function of incubation in simulated body fluid (gastric and salivary) together with graphical representations showing the changes in surface topography.

To quantify the surface changes caused by the degradation, the surface roughness was evaluated using a confocal microscope. The changes in *R*_a_ values (representing surface roughness) for the samples after *in vitro* tests are summarized in Figure 11. As can be inferred from the nonmonotonous changes of *R*_a_ with time, there are several individual processes involved in the overall degradation mechanism. In the first stage (first 2 weeks) of degradation, the pores expand, they become deeper, and their walls become thinner, which is reflected in a substantial increase in the *R*_a_ value. When the degradation process prolongs, *R*_a_ decreases due to the salt precipitation in the pores and the destruction of the walls. In line with the SEM observations and spectroscopic data (Figures 5, 6, 9, and 10), the changes in roughness clearly show the differences in the degradation process under two *in vitro* conditions (saliva, gastric), mostly manifested after a longer time. Surface irregularities play an important role in the tissue–implant interface. The increase in roughness and related increase in the surface area available for tissue overgrowth may accelerate restenosis, as observed in clinical practice (J.W. and J.K., private information).

**Figure 12:**
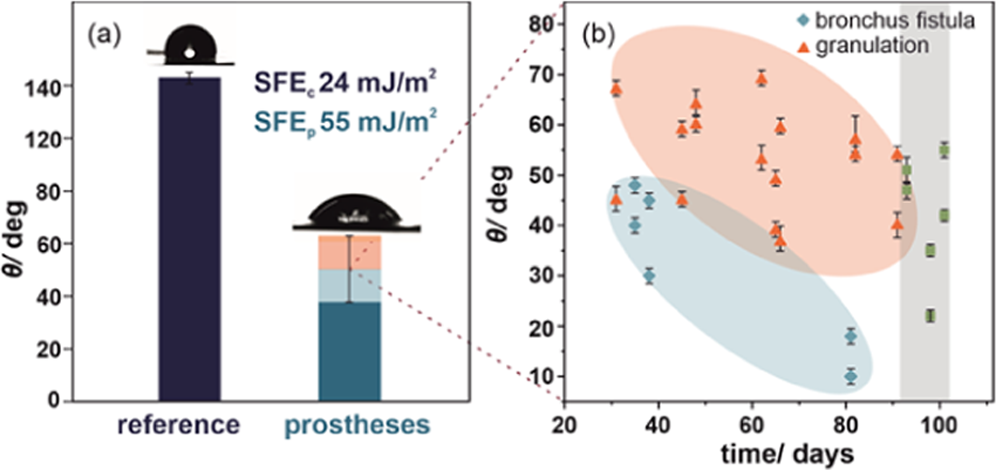
Representative results of water contact angle measurements and the corresponding surface free energy for the investigated SEMS polyurethane covers (a): reference (navy blue) and implanted prostheses (blue). The shadowing illustrates the range of experimental values for the investigated samples. Detailed analysis of water contact angle measurements as a function of time in the human body (b).

Additional surface characterization was provided by the water contact angle measurements; this method quantitatively characterizes the wettability and allows for surface free energy (SFE) determination, often used for evaluation of the biological interactions such as adhesion of proteins, cells, or tissues. The exposure of the esophageal stent to human body fluids causes significant changes in the surface properties of the polymeric membrane. The polyurethane cover of the esophageal stent is initially hydrophobic, as revealed by the water contact angle of 123 ± 2° and the corresponding SFE = 24 mJ/m^2^ for the reference material. The changes induced by prolonged interaction with body fluids strongly affect the examined prostheses with a significant decrease in average water contact angle down to 49 ± 15° (Figure 12). This can be explained by changes in the surface morphology, especially degradation and physical damages observed in SEM images and *R*_a_ (Figures 7–11). Nevertheless, it is worth underlying that although the SEM observations provide local information about the degradation, the contact angle measurements characterize the macroscopic changes at the polymeric surface.

As can be observed in Figure 12b, independently the type of complications (granulation, bronchus fistula), the overall trend in contact angle changes is the same: the longer the implantation time, the smaller the contact angle value. The observed trend indicates that polymer degradation is stable within the investigated range of implantation time. The gray shadowing corresponds to the time recommended by the implant producer and was achieved only for stents without complications. It can also be noted that in the case of bronchus fistula, the water contact angle values were lower than those for granulation.

The various methods of the prostheses’ structure examination show the different character of the changes in the distal and proximal segments of the prostheses’ polymer. The structural changes result from the influence of the applied chemo- and radiotherapy treatment, mechanical stress, as well as the influence of the esophagus and gastric contents. Since the major damage was also observed for the prosthesis not exposed to chemo- or radiotherapy, the surrounding environment of the SEMS is a sufficient factor to elicit significant surface and structural changes in the polyurethane membrane.^53,54^ The SEM/CM observations confirmed that the destruction processes are quite extensive and interlaced, resulting in etching and peeling off of the material. The observed crack formation and propagation on the surface of the polyurethane are mainly connected with the high mechanical stress generated by nitinol mesh as well as the surrounding tissue pressure. Thus, it can be concluded that the applied polyurethane material has insufficient mechanical strength resistance for prolonged use in the body.

The chemical degradation of polyurethanes is mainly caused by hydrolysis as thoroughly discussed elsewhere.^55,56^ This type of degradation is a combination of *in vivo* chemical factors such as water sorption, pH, presence of radicals, and biological moieties like cells, enzymes, and lipids. Since the water molecules mostly interact with the polyurethane polar groups, the hydrolytic degradation is dominating and affects carbonyl bands (changes in IR spectra at 1730 cm^–1^ in Figures 5 and 6). As shown in Figures 9–11, the interaction with simulated body fluids already leads to surface damages and microscopic cracking. However, as reported for *in vivo* conditions,^57^ biological factors (i.e., FBGC, enzymes) accelerate the hydrolytic degradation, which can be additionally enhanced by mechanical stress. It is also known that the cracks initiated by chemical interaction can be propagated by residual stress. This in turn causes an increase in the surface area and the number of available surface sites that accelerate the hydrolytic degradation even further.^58^ It is worth mentioning that the biological degradation is diffusion-limited and thus confined to a few microns and thus can be considered as a mostly surface process. It can be thus concluded that although the cracks may open the diffusion pathways for bulk degradation, the obtained results (SEM, IR, CM–*R*_a_) indicate that the polyurethane degradation process begins at the surface. Such results are in good agreements with the previously reported *in vivo* investigations.^51^

In the studied group, it was found that among patients with fistula, the polymeric surface was exposed to body fluids for a shorter time (35–81 days) than in the group with granulation (31–153 days). The mechanism of polymer degradation can be one of the factors leading to a life-threatening complication, which is a fistula to the bronchial tree. The formation of granulation tissue after the implantation of the esophageal prosthesis is a typical complication occurring in the range of 4–40%^59,60^ (in our work, 11%). It is formed in the distal and proximal ends of the prostheses in healthy tissues of the esophagus as well. The granule is a noncancerous connective tissue that surpasses the prosthetic ends most likely due to the exudation of the mechanical stress of the nitinol mesh on the esophageal mucosa.^61,62^ Based on our operating room observations, such an effect can be caused by a lack of SEMS nitinol biomechanical compatibility causing irradiation of the esophageal wall, likely causing granulation. Moreover, the irradiation at the esophageal wall–prosthesis interface is enhanced by the progressive degradation of the polymer bulk and surface. The granulation tissue grows up to about 3 months after the prosthesis implantation, leading to complete obstruction of the esophageal stent, which in turn leads to endoscopic removal of the prosthesis, already at high risk of complications. The mechanism of granulation formation may be also related to the destructive effect of acid gastric contents, especially in its distal segment.

The influence of prosthetics, irradiation, and chemotherapy on the esophagus and the occurrence of complications after stenting in the course of cancer remain unclear. The observed changes in the microstructure of the esophagus and secondary changes in the esophageal wall are important for the pathophysiology. Factors determining their occurrence are yet to be investigated, and the presented report clearly shows a need and direction for further research. The degradation of polyurethanes is mostly examined *in vitro*, and in many cases in stable environments under favorable temperature conditions.^63,64^ Such experiments performed under controllable conditions do not provide thorough feedback of the material in such a complex system as the human body. Within this study, we address this knowledge gap. The in-depth insights are the prerequisites for the development of new-generation prostheses with better parameters, which can minimize the traumatic impact of the foreign body on the esophagus.

The performed studies clearly point out the important practical implications and the need for different optimization of the polymeric material, specifically for each end of the stent. The hydrophilic/hydrophobic polymeric surfaces at the outer and inner sides of the implant will be beneficial to provide biocompatibility and prevent obstruction, respectively. Such modification can be obtained using plasma treatment (introduction of surface functional groups such as −OH or −F without changing the bulk properties). Another strategy consists of improving the chemical inertness and mechanical properties by applying polymeric composites with tunable properties.

## Conclusions

In this work, we have evaluated the effect of the human body environment and the applied cancer treatment on the degradation process of the polyurethane SEMS membranes. The obtained physicochemical characterization of 16 stents removed from the patients revealed that independent of the treatment (chemo- and/or radiotherapy, none), all of the polymeric membranes systematically degrade *in vivo* as a function of implantation times (31–153 days). The differences between the proximal and distal ends were identified and explained in terms of various chemical natures of the body fluids. This study was supported by the parallel *in vitro* tests in simulated body fluids representative for gastric and saliva environment. These findings have important practical implications, pointing out the need for different optimization of the material properties, specifically for each end of the stent. The improvement of the chemical stability of the polymeric material is of importance, since even a short implantation time (35 days) led to significant changes in the surface and bulk structure, as observed by DSC, DMTA, ATR-IR, SEM, CM, and CA methods. The obtained results also show that the contact angle and the polymer melting temperature can be considered as suitable parameters for analyzing the extent of the stent degradation processes. To the best of our knowledge, this work is the only report in the literature that shows the influence of chemo- and radiotherapy and the role of the microenvironment of the esophagus and stomach in the structure of the polyurethane/nitinol stents.

## Supporting Information Available

Scheme of esophageal stent with proximal release, typical image used for evaluation of polyurethane membrane surface roughness, and detailed SEM images of surface degradation for *in vitro* experiment in artificial body fluid (PDF)
