# Supplementary material for: Characterization of Partially Covered Self-Expandable Metallic Stents for Esophageal Cancer Treatment: In Vivo Degradation
Source: ACS Biomater Sci Eng. 2021 Mar 12;7(4):1403–13. doi: 10.1021/acsbiomaterials.0c01773 (PMC8045022; doi:10.1021/acsbiomaterials.0c01773)
Supplement: Supplementary file 1 — ab0c01773_si_001.pdf [file ab0c01773_si_001.pdf]

Characterization of partially covered self-expandable metallic stents for esophageal cancer treatment: in vivo degradation

Paulina Chytrosz[1], Monika Golda – Cepa[1], Janusz Wlodarczyk[2], Jarosław Kuzdzal[2], Miroslawa El Fray[3], Andrzej Kotarba[1*]*

[1] Faculty of Chemistry, Jagiellonian University, Kraków, Poland

[2] Department of Thoracic and Surgical Oncology, Jagiellonian University Medical College, John Paul II Hospital, Kraków, Poland

[3] Department of Polymer and Biomaterials Science, West Pomeranian University of Technology, Szczecin, Poland

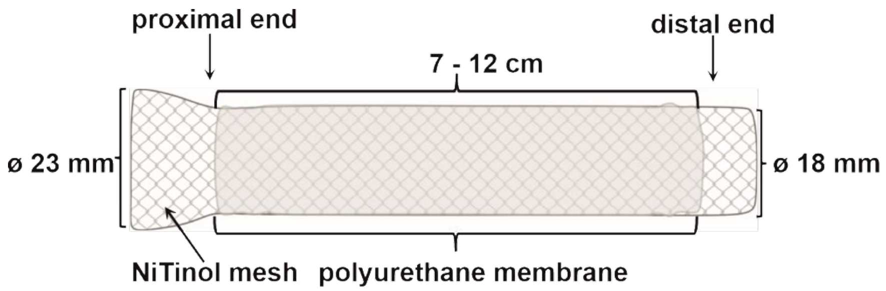

Figure SI.1. Scheme of esophageal stent with proximal release used in this study.

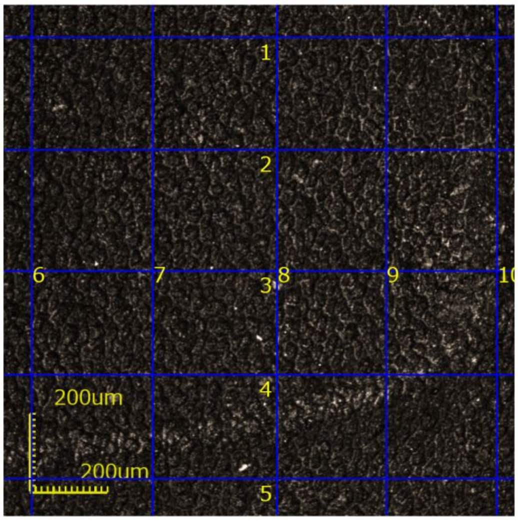

Figure SI.2. Typical image used for evaluation of polyurethane membrane surface roughness.

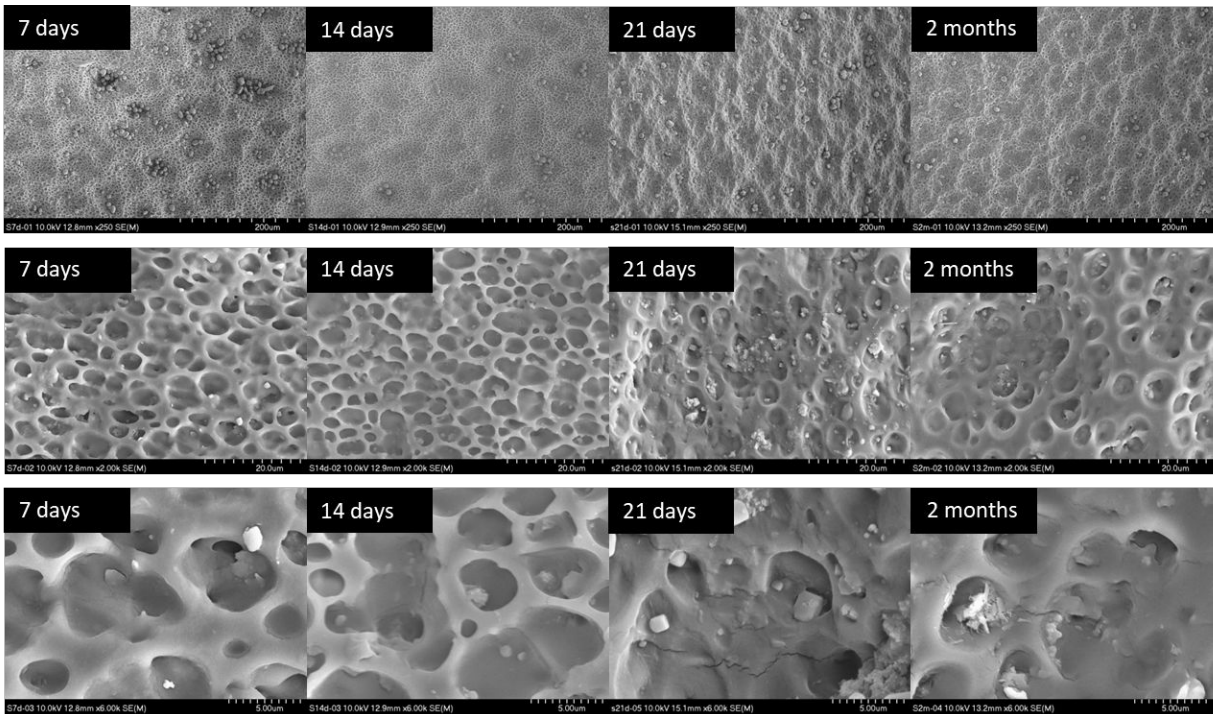

Figure SI.3. Detailed SEM images of surface degradation for *in vitro* experiment in artificial saliva (inner side of the stent).

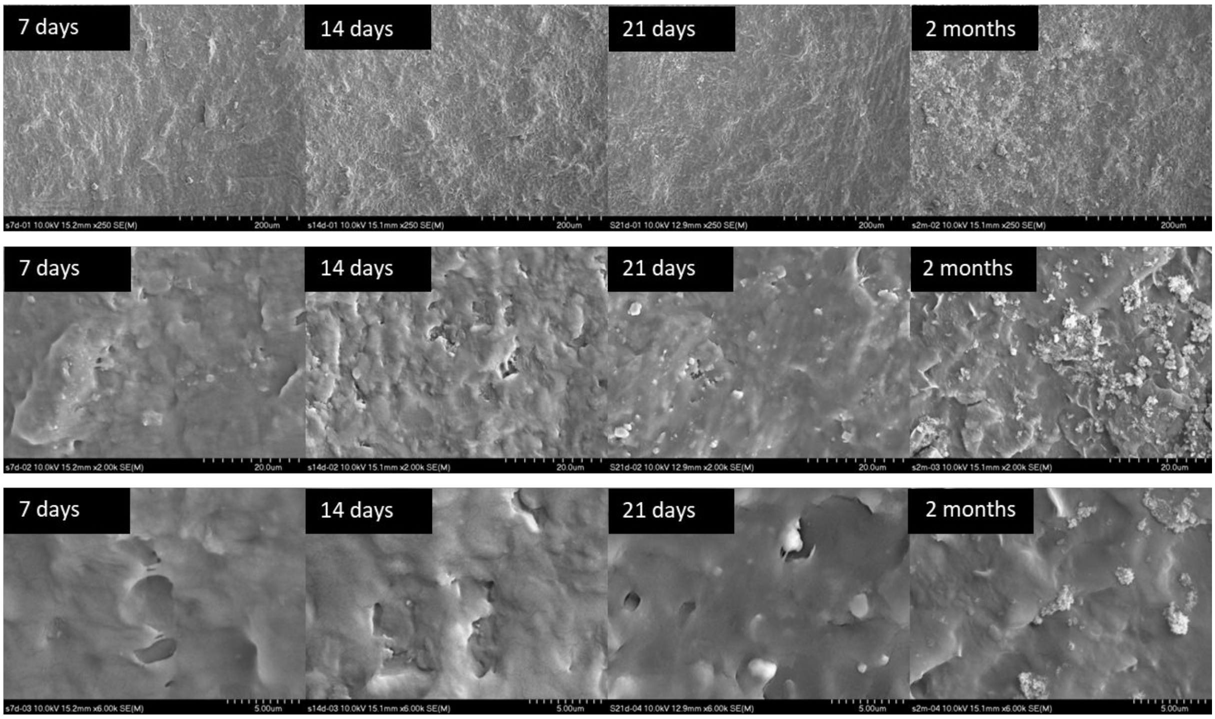

Figure SI.4. Detailed SEM images of surface degradation for *in vitro* experiment in artificial saliva (outer side of the stent).

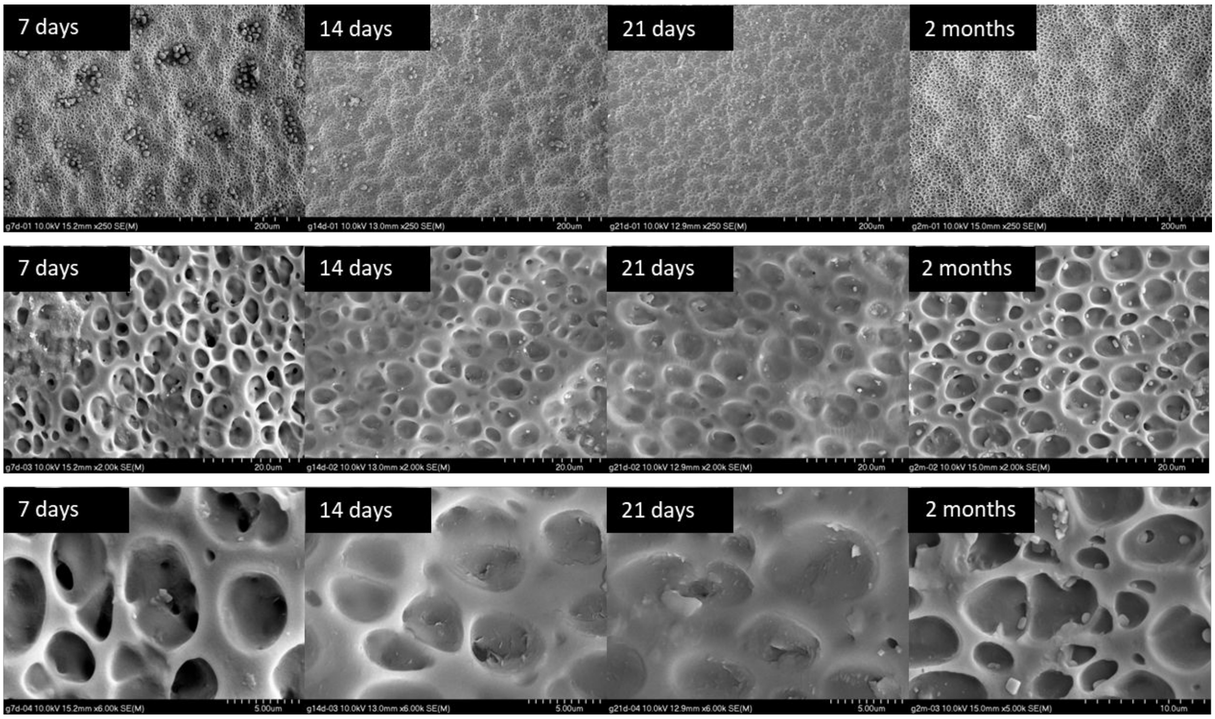

Figure SI.5. Detailed SEM images of surface degradation for *in vitro* experiment in simulated gastric fluid (inner side of the stent).

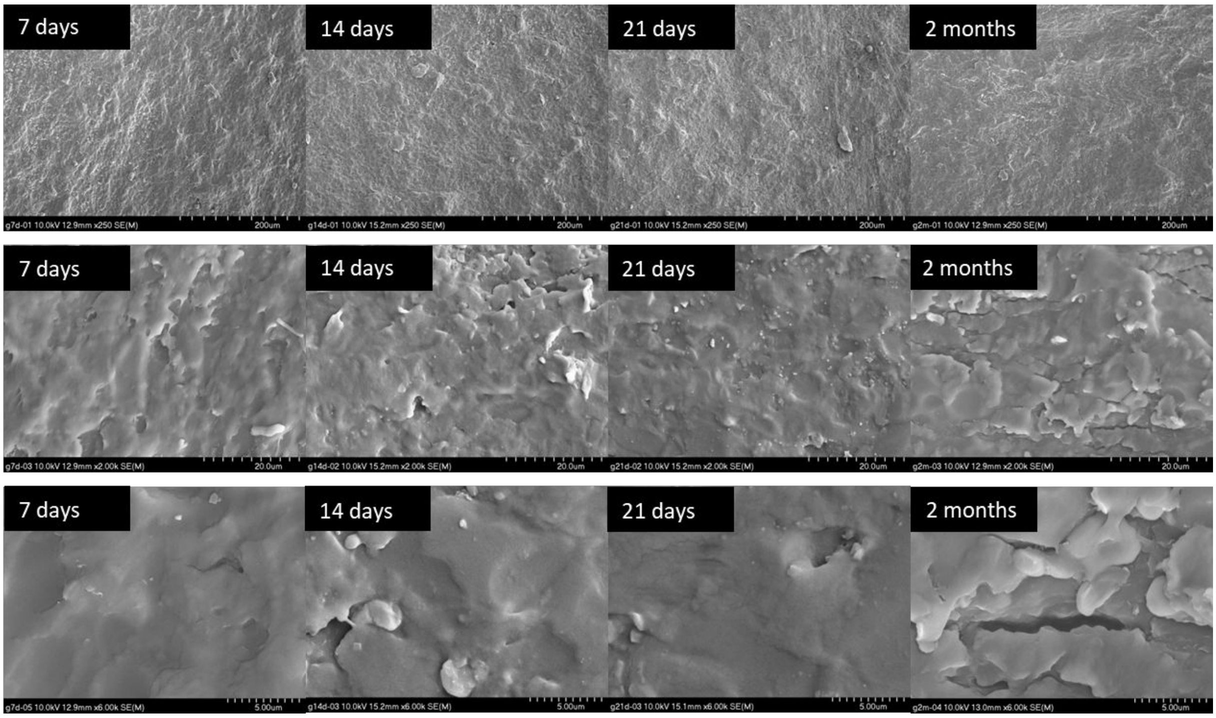

Figure SI.6. Detailed SEM images of surface degradation for *in vitro* experiment in simulated gastric fluid (outer side of the stent).
